# Clinical Characteristics, Treatment Approaches, and Survival Predictors in Adult Acute Myeloid Leukemia: Interim Results from the Turkish Society of Hematology AML Registry †

**DOI:** 10.3390/jcm14207367

**Published:** 2025-10-18

**Authors:** Volkan Karakus, Ibrahim Ethem Pinar, Utku Iltar, Emel Merve Yenihayat, Merve Gokcen Polat, Serhat Celik, Umit Yavuz Malkan, Guldane Cengiz Seval, Ali Dogan, Aydan Akdeniz, Demircan Ozbalci, Idris Ince, Ramazan Erdem, Ozgur Mehtap, Hakki Onur Kirkizlar, Murat Kacmaz, Burak Deveci, Fatma Aykas, Gulten Korkmaz, Sureyya Yigit Kaya, Hacer Berna Afacan Ozturk, Omur Gokmen Sevindik, Ferda Can, Demet Cekdemir, Ceyda Aslan, Hale Bulbul, Zeynep Tugba Karabulut, Senem Maral, Salih Sertac Durusoy, Fatih Demirkan, Hakan Goker, Fahir Ozkalemkas, Muzaffer Keklik, Selami Kocak Toprak, Aylin Fatma Karatas, Unal Atas, Inci Alacacioglu

**Affiliations:** 1 Department of Hematology, University of Health Sciences, Antalya Training and Research Hospital, 07100Antalya, Turkey; 2 Department of Hematology, Faculty of Medicine, Bursa Uludag University, 16059 Bursa, Turkey; 3 Department of Hematology, Faculty of Medicine, Akdeniz University, 07070 Antalya, Turkey; 4 Department of Hematology, Faculty of Medicine, Kocaeli University, 41001 Kocaeli, Turkey; 5 Department of Hematology, Faculty of Medicine, Erciyes University, 38030 Kayseri, Turkey; 6 Department of Hematology, Faculty of Medicine, Hacettepe University, 06230 Ankara, Turkey; 7 Department of Hematology, Faculty of Medicine, Ankara University, 06230 Ankara, Turkey; 8 Department of Hematology, Faculty of Medicine, Van Yuzuncu Yil University, 65080 Van, Turkey; 9 Department of Hematology, Faculty of Medicine, Mersin University, 33343 Mersin, Turkey; 10 Department of Hematology, Faculty of Medicine, Suleyman Demirel University, 32260 Isparta, Turkey; 11 Department of Hematology, Gaziantep Dr. Ersin Arslan Training and Research Hospital, 27090 Gaziantep, Turkey; 12 Department of Hematology, Faculty of Medicine, Trakya University, 22030 Edirne, Turkey; 13 Department of Hematology, Faculty of Medicine, Hatay Mustafa Kemal University, 31001 Hatay, Turkey; 14 Stem Cell Transplant Unit, Department of Hematology, Medstar Antalya Hospital, 07050 Antalya, Turkey; 15 Department of Hematology, University of Health Sciences, Ankara City Hospital, 06800 Ankara, Turkey; 16 Department of Hematology, Manisa City Hospital, 45040 Manisa, Turkey; 17 Department of Hematology, University of Health Sciences, Diskapi Yildirim Beyazit Training and Research Hospital, 06110 Ankara, Turkey; 18 Department of Hematology, International School of Medicine, Istanbul Medipol University, 34214 Istanbul, Turkey; 19 Department of Hematology, University of Health Sciences, Tepecik Training and Research Hospital, 35020 Izmir, Turkey; 20 Department of Hematology, University of Health Sciences, Derince Training and Research Hospital, 41900 Kocaeli, Turkey; 21 Department of Hematology, Nigde Omer Halisdemir University Training and Research Hospital, 51100 Nigde, Turkey; 22 Department of Hematology, School of Medicine, Sanko University, 27090 Gaziantep, Turkey; 23 Department of Hematology, Faculty of Medicine, Dokuz Eylul University, 35340 Izmir, Turkey

**Keywords:** acute myeloid leukemia, real-world data, prospective registry, personalized therapy, treatment intensity, survival predictors, ECOG performance status, ELN risk classification

## Abstract

**Background**: Acute myeloid leukemia (AML) is an aggressive and biologically diverse hematologic cancer that disproportionately affects older individuals. Despite advances in molecular profiling and therapy, long-term outcomes remain unsatisfactory. This nationwide registry was established to provide real-world insights into clinical characteristics, treatment strategies, and survival among adult AML patients in Turkey. **Methods**: The Turkish AML Registry Project (ClinicalTrials.gov Identifier: NCT05979675) combines retrospective and prospective data from 23 tertiary hematology centers. Adult patients diagnosed between January 2008 and July 2023 were included. Baseline demographics, European LeukemiaNet (ELN) 2017 risk groups, Eastern Cooperative Oncology Group (ECOG) performance status, treatment intensity, and targeted therapy use were analyzed. Response and survival outcomes were assessed using Kaplan–Meier methods. **Results**: The interim dataset included 891 patients (median age 58 years, 45.5% ≥60). Intensive chemotherapy, most commonly 7 + 3, was applied in 74.1%, while 25.9% received lower-intensity regimens. Targeted agents, mainly venetoclax, were incorporated more frequently into low-intensity therapies (19.1% vs. 3.4%, *p* < 0.001). Complete remission occurred in 70.2% after intensive and 35.9% after low-intensity therapy, improving to 51.4% with targeted agents. Median overall survival (OS) was 27.2 months, with 1-year OS rates of 54.1%, 28.9%, and 17.6% for favorable, intermediate, and adverse ELN groups (*p* < 0.001). ECOG 0–1 predicted superior survival (1-year OS 70.3% vs. 47.0%). **Conclusions**: Nationwide real-world evidence underscores the prognostic relevance of ELN risk and functional status in AML. While intensive chemotherapy remains central, combining targeted agents with low-intensity regimens improves outcomes in less fit patients and supports personalized treatment approaches.

## 1. Introduction

Acute myeloid leukemia (AML) represents a biologically heterogeneous yet clinically aggressive myeloid neoplasm, driven by uncontrolled expansion of immature progenitor cells and most commonly encountered in older individuals [1,2]. Despite therapeutic advances, prognosis remains poor in elderly and high-risk populations, with 5-year survival rates declining steeply with age [3,4].

Over the past three decades, both the incidence and mortality of AML have increased globally, particularly among older adults [5,6]. In high-income regions such as North America and Western Europe, improved diagnostic capacity has revealed higher incidence but better outcomes, whereas survival rates remain markedly lower in low- and middle-income countries [5,7]. Among patients aged ≥60 years, 5-year survival is generally below 15%, compared with approximately 50% in younger adults [6].

Standard AML management involves induction chemotherapy—typically the intensive ‘7 + 3’ regimen—followed by consolidation with high-dose cytarabine or allogeneic hematopoietic stem cell transplantation (HSCT) in eligible cases [1,2,4,8]. For patients unfit for intensive treatment, non-intensive regimens with or without targeted therapies are used as disease-modifying approaches [1,2,8,9,10].

However, access to intensive chemotherapy, molecular diagnostics, and novel targeted agents remains heterogeneous worldwide, particularly in middle-income healthcare systems [11]. The integration of targeted therapies such as venetoclax or fms-like tyrosine kinase 3 (*FLT3*) inhibitors has improved remission and survival rates in unfit and older patients, yet these agents remain unavailable to many due to cost and reimbursement barriers [12,13,14].

Although complete remission (CR) is achieved in up to 70–80% of fit patients after induction, relapse or refractory disease remains common [10,15,16]. Outcomes are worse for patients receiving non-intensive treatments, with most unable to achieve durable remissions [15,16,17,18].

Moreover, disease biology—including cytogenetic and molecular risk—significantly influences prognosis and treatment response [1,3,4,8,19,20,21,22,23].

Fitness assessment remains subjective despite existing scoring systems [24], and global disparities persist in treatment accessibility due to variations in clinical practice, reimbursement, and regulatory frameworks [25].

While clinical trials have shaped current AML treatment standards, real-world outcomes often differ substantially due to broader patient heterogeneity and healthcare variability. Large-scale population-based registries from Europe and Asia have revealed lower remission and survival rates in unselected populations, driven by older age, comorbidities, and limited transplant access [7,26,27]. However, prospective real-world evidence from developing or middle-income healthcare systems remains scarce. National AML registries play a pivotal role in bridging this evidence gap by demonstrating how international guidelines are implemented in routine clinical practice under heterogeneous healthcare conditions [28].

In this context, establishing a national AML registry represents a crucial step toward generating real-world evidence in Turkey. Designed as a nationwide initiative, the Turkish AML Registry Project integrates data from multiple treatment environments to reflect real-world management practices. The present interim evaluation provides an overview of demographic and clinical profiles, therapeutic patterns, and one-year outcomes among adults diagnosed with AML in Turkey. In light of expanding targeted therapy options [1,29,30,31,32,33,34], this study highlights critical associations between treatment intensity, Eastern Cooperative Oncology Group (ECOG) performance status, European LeukemiaNet (ELN) risk classification, and survival outcomes, aiming to inform context-specific, personalized care strategies in AML.

## 2. Methods

### 2.1. Study Design and Population

This study is a nationwide, multicenter, non-interventional observational registry combining retrospective and prospective data collection (ClinicalTrials.gov Identifier: NCT05979675), conducted across 23 tertiary hematology centers in Turkey.

The retrospective phase included adult patients diagnosed with AML between January 2008 and June 2022, with anonymized data collected from institutional medical records, as approved by the Istanbul Medipol University Non-Interventional Clinical Research Ethics Committee (Approval Date: 3 September 2021, Protocol No: E-108400098-772.02-4214).

The prospective phase was launched in June 2022 and is designed for a 5-year follow-up period (June 2022–June 2026). This phase received approval from the Istanbul Medipol University Clinical Research Ethics Committee (Approval Date: 21 February 2022, Protocol No: E-66291034-772.02-1237) and from the Republic of Turkey Ministry of Health Turkish Medicines and Medical Devices Agency (Approval Date: 14 April 2022, Protocol No: 202105019).

The present interim analysis includes baseline characteristics and 1-year outcomes of retrospectively and prospectively enrolled patients, with data collected up to July 2023. Survival analyses accounted for variable follow-up durations through right-censoring.

A total of 906 adult patients diagnosed with AML were initially identified. After excluding 15 patients due to incomplete clinical data, 891 patients were included in the final analysis. The median age at diagnosis was 58 years (range, 18–91), and 54.9% were male.

The study was conducted in accordance with the Declaration of Helsinki, and written informed consent was obtained from all prospectively enrolled participants.

Genomic analyses were performed locally at participating institutions without central review, reflecting real-world diagnostic practice.

### 2.2. Data Collection and Definitions

Baseline demographic and clinical data included age, gender, AML subtype (according to the World Health Organization classification, WHO), ECOG performance status, ELN 2017 genetic risk classification [19], presence of extramedullary disease, and molecular status for FLT3-internal tandem duplication (*FLT3-ITD*) and nucleophosmin 1 (*NPM1*) mutations.

The treating physician determined the ECOG performance status at the time of AML diagnosis. Cytogenetic and molecular evaluations were carried out using bone marrow aspirates or, when applicable, peripheral blood samples obtained before initiation of induction therapy. Molecular analyses relied on institution-specific polymerase chain reaction (PCR) assays or next-generation sequencing (NGS) panels that had undergone local validation, whereas chromosomal abnormalities were identified through conventional karyotyping and fluorescence in situ hybridization (FISH) according to standard laboratory procedures.

Molecular analyses were conducted according to the diagnostic capacity of each participating center. *FLT3* and *NPM1* were the most frequently assessed markers, while additional genes—including *IDH1/2*, *TP53*, *RUNX1*, *ASXL1*, *CEBPA*, *KIT*, and *KMT2A*—were tested in centers equipped with PCR or NGS facilities. Cytogenetic abnormalities such as t(8;21), inv(16)/t(16;16), t(15;17), del(17p), and other recurrent aberrations were identified by conventional karyotyping, FISH, or narrative cytogenetic reports. Given the real-world nature of the registry, testing coverage varied across centers and over time; missing molecular data were not imputed.

Treatment-related variables included induction regimen intensity (high-intensity vs. low-intensity), use of targeted agents (e.g., venetoclax, midostaurin, gemtuzumab ozogamicin, sorafenib). The distribution of targeted agents across treatment intensities is presented in Supplementary Figure S1.

Treatment response was categorized as CR, partial response (PR), morphologic leukemia-free state, stable disease (SD), or progressive disease (PD), based on ELN 2017 response criteria. Overall survival (OS) represented the interval between initial diagnosis and either death from any cause or the date of last contact. Progression-free survival (PFS) was calculated from diagnosis until documented relapse, disease advancement, or death, whichever occurred first.

### 2.3. AML Subtypes and Risk Stratification

AML subtypes were classified according to the 2016 WHO criteria [8,35]. Risk stratification was performed in accordance with the 2017 ELN recommendations, categorizing patients into favorable, intermediate, and adverse genetic risk groups [19,36]. ELN categories were assigned using the available molecular and cytogenetic results for each patient, consistent with real-world registry practice. Missing molecular data were not imputed to preserve data integrity.

### 2.4. Statistical Analysis

Baseline characteristics were analyzed through descriptive statistical methods. Categorical variables were summarized as absolute frequencies and percentages, whereas continuous variables were described using medians with their corresponding ranges. Associations between categorical parameters were examined with the chi-square (χ^2^) test, and non-parametric group differences were evaluated using the Mann–Whitney U test.

Survival probabilities were estimated by the Kaplan–Meier approach, and intergroup differences were compared using the log-rank test. Where appropriate, pairwise and multivariate log-rank analyses were additionally conducted. Statistical significance was determined at a two-sided *p* value threshold of 0.05.

Analyses were conducted using Python (v3.13.2; Clang 16.0.0) in a virtual environment on macOS. Data preprocessing and transformation were performed with pandas, while survival modeling and statistical plotting utilized lifelines, matplotlib, and scipy.

## 3. Results

### 3.1. Patient Characteristics

The analysis encompassed 891 adults diagnosed with AML. Patients had a median age of 58 years (range 18–91), and slightly more than half of the cohort (54.9%) were men. According to WHO classification, AML not otherwise specified was the most prevalent subtype (55.8%), followed by AML with recurrent genetic abnormalities (17.6%) and AML with myelodysplasia-related changes (15.4%) (Table 1). Extramedullary involvement was documented in 4.4% of patients. Based on the 2017 ELN risk classification, 24.7% of patients were categorized as favorable-risk, 60.8% as intermediate-risk, and 14.5% as adverse-risk. At diagnosis, 63.1% of patients had an ECOG performance status of 0–1, while 36.9% were ECOG ≥ 2. In patients for whom molecular results were available, FLT3-ITD mutations were identified in 15.7% and NPM1 mutations in 20.1% of cases.

Beyond *FLT3-ITD* and *NPM1*, additional molecular and cytogenetic analyses were performed according to the diagnostic capacity of participating centers. *IDH1/2*, *TP53*, *RUNX1*, *ASXL1*, *CEBPA*, *KIT*, and *KMT2A* mutations were assessed in a subset of patients with available PCR or NGS testing, while recurrent cytogenetic abnormalities such as t(8;21), inv(16)/t(16;16), t(15;17), and del(17p) were detected by karyotyping, FISH, or narrative cytogenetic reports.

The frequency of tested patients and detected abnormalities for each marker is summarized in Supplementary Table S1, which reflects real-world heterogeneity in genomic testing coverage across the 23 centers.

### 3.2. Treatment Distribution and Targeted Therapy Use

**Figure 1:**
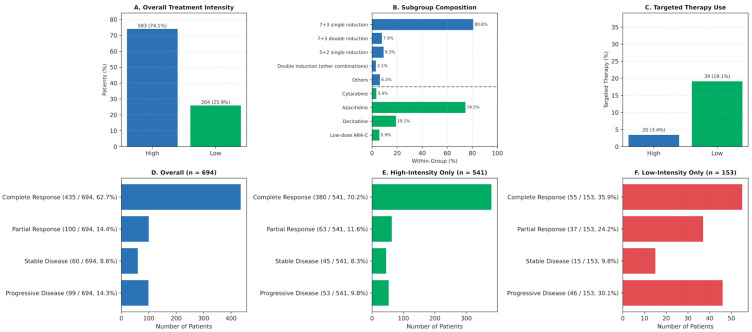
Treatment intensity, regimen composition, targeted therapy use, and response rates in the study cohort. (**A**) Overall distribution of treatment intensity: high-intensity versus low-intensity induction regimens. (**B**) Composition of specific induction regimens within each treatment intensity group. (**C**) Proportion of patients receiving targeted therapy stratified by treatment intensity. (**D**) Overall response rates (complete remission [CR], partial response [PR], stable disease [SD], progressive disease [PD]) among all evaluable patients (n = 694). (**E**) Response rates among patients receiving high-intensity therapy (n = 541). (**F**) Response rates among patients receiving low-intensity therapy (n = 153). Abbreviations: CR, complete remission; PR, partial response; SD, stable disease; PD, progressive disease.

Of the cohort, 74.1% received high-intensity induction therapy, while 25.9% were treated with low-intensity regimens (Figure 1A). Among high-intensity protocols, 7 + 3 single induction was dominant (80.8%), whereas azacitidine was the leading agent in low-intensity regimens (74.5%), followed by decitabine (19.1%) (Figure 1B). Targeted therapies were more frequently administered in patients receiving low-intensity regimens (19.1%) than those on high-intensity protocols (3.4%) (*p* < 0.001; Figure 1C), reflecting a clinical preference for integrating targeted agents in older or frail individuals. Subgroup distribution by regimen is shown in Supplementary Figure S1.

### 3.3. Response Rates

Treatment response differed significantly by intensity (*p* < 0.001). CR was achieved in 70.2% of patients on high-intensity regimens compared to 35.9% of those receiving low-intensity therapies. PD occurred in 30.1% of low-intensity patients versus 9.8% in the high-intensity group. PR and SD rates also favored high-intensity therapy (11.6% vs. 24.2% for PR; 8.3% vs. 9.8% for SD) (Figure 1E,F).

**Figure 2:**
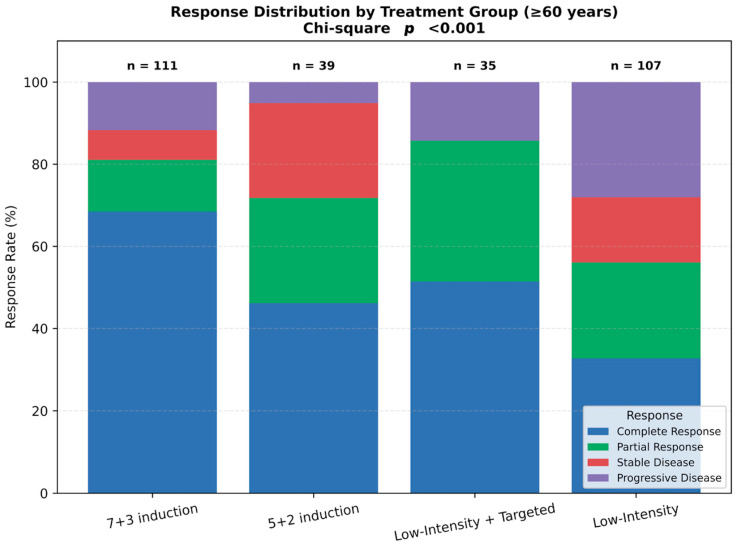
Response distribution by treatment group among patients aged ≥60 years. Stacked bar plot showing response rates—complete remission (CR), partial response (PR), stable disease (SD), and progressive disease (PD)—according to induction regimen: 7 + 3 induction, 5 + 2 induction, low-intensity therapy with targeted agents, and low-intensity therapy without targeted agents. Significant differences in response distributions were observed across groups (Chi-square test, *p* < 0.001). Abbreviations: CR, complete remission; PR, partial response; SD, stable disease; PD, progressive disease.

Stratifying by both intensity and targeted agent use, marked differences in response were observed (Figure 2). In the low-intensity group, adding targeted therapy improved CR rates from 35.9% to 51.4% and halved the PD rate from 30.1% to 14.3%. A similar but less pronounced benefit was seen in the high-intensity cohort. These differences were statistically significant (*p* < 0.001), emphasizing the additive impact of targeted therapy on disease control.

**Figure 3:**
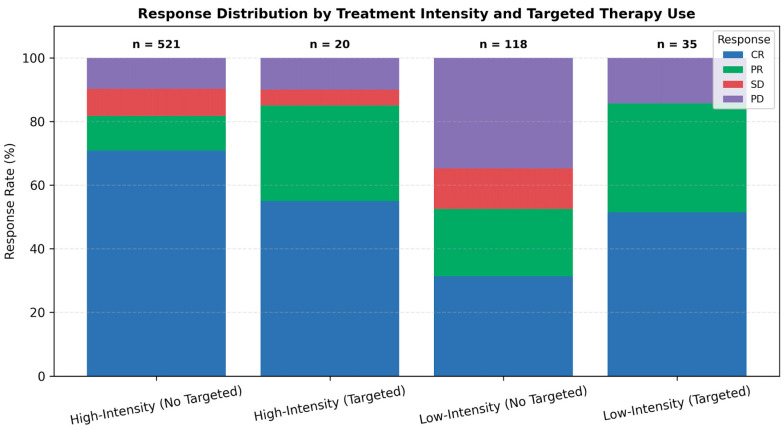
Response distribution by treatment intensity and targeted therapy use. Stacked bar plot showing response rates—complete remission (CR), partial response (PR), stable disease (SD), and progressive disease (PD)—according to treatment intensity (high-intensity vs. low-intensity) and targeted therapy use (targeted vs. no targeted agents). Addition of targeted therapy was associated with improved response profiles across both treatment intensity groups. Abbreviations: CR, complete remission; PR, partial response; SD, stable disease; PD, progressive disease.

In participants aged ≥60 years, response distributions varied significantly across regimens (*p* < 0.001; Figure 3). The 7 + 3 induction group demonstrated the highest CR rate, while low-intensity plus targeted therapy yielded favorable response profiles with minimal progression. In contrast, low-intensity non-targeted regimens were associated with the highest rates of PD and SD. These findings highlight how treatment strategies are individualized according to patient age and fitness in real-world clinical practice.

### 3.4. Survival Outcomes

**Figure 4:**
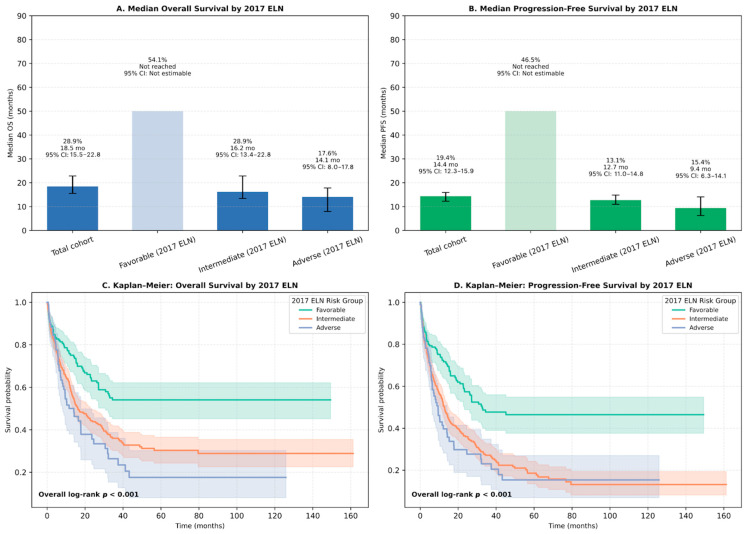
Overall survival (OS) and progression-free survival (PFS) by 2017 ELN risk classification. (**A**) Median OS and 1-year OS rates stratified by ELN 2017 risk groups (favorable, intermediate, adverse) compared to the total cohort. (**B**) Median PFS and 1-year PFS rates stratified by ELN 2017 risk groups compared to the total cohort. (**C**) Kaplan–Meier curves illustrating OS by ELN 2017 risk groups, with significant differences across groups (overall log-rank *p* < 0.001). (**D**) Kaplan–Meier curves illustrating PFS by ELN 2017 risk groups, with significant differences across groups (overall log-rank *p* < 0.001). Abbreviations: OS, overall survival; PFS, progression-free survival; ELN, European LeukemiaNet.

Outcomes stratified by ELN 2017 classification revealed substantial differences. Patients in the favorable-risk group had a 1-year OS rate of 54.1%, with median OS not reached (95% CI: 30.8–NA), whereas intermediate- and adverse-risk patients had 1-year OS rates of 28.9% and 17.6%, and median OS of 16.2 months (95% CI: 13.4–22.8) and 14.1 months (95% CI: 8.0–17.8), respectively (log-rank *p* < 0.001; Figure 4A–C). Similarly, 1-year PFS was 46.5% in favorable-risk patients (median PFS: 32.6 months), compared to 13.1% and 15.4% in intermediate- and adverse-risk groups (median PFS: 12.7 and 9.4 months, respectively) (log-rank *p* < 0.001; Figure 4B–D).

**Figure 5:**
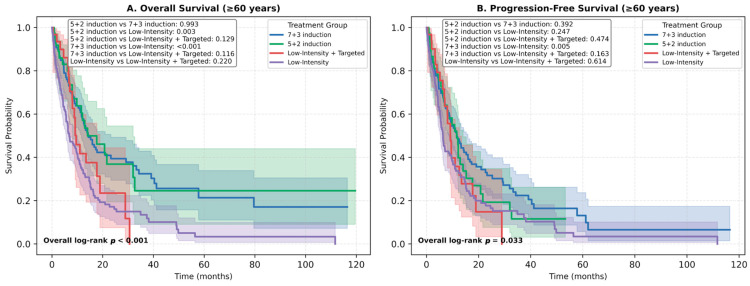
Overall survival (OS) and progression-free survival (PFS) by treatment regimen among patients aged ≥60 years. (**A**) Kaplan–Meier curves showing OS according to treatment groups: 7 + 3 induction, 5 + 2 induction, low-intensity therapy with targeted agents, and low-intensity therapy without targeted agents. (**B**) Kaplan–Meier curves showing PFS according to the same treatment groups. Overall survival differed significantly across groups (log-rank *p* < 0.001), while progression-free survival differences were more modest (log-rank *p* = 0.033). Pairwise *p*-values between treatment groups are provided within each panel. Abbreviations: OS, overall survival; PFS, progression-free survival.

Among elderly patients (≥60 years), 1-year OS rates were 54.2% for 7 + 3 induction, 52.3% for 5 + 2 induction, and 48.5% for low-intensity targeted therapy, compared to only 31.4% for non-targeted low-intensity regimens. Median OS ranged from 17.6 months in the 7 + 3 group to 8.6 months in the low-intensity non-targeted group (Figure 5A,B).

**Figure 6:**
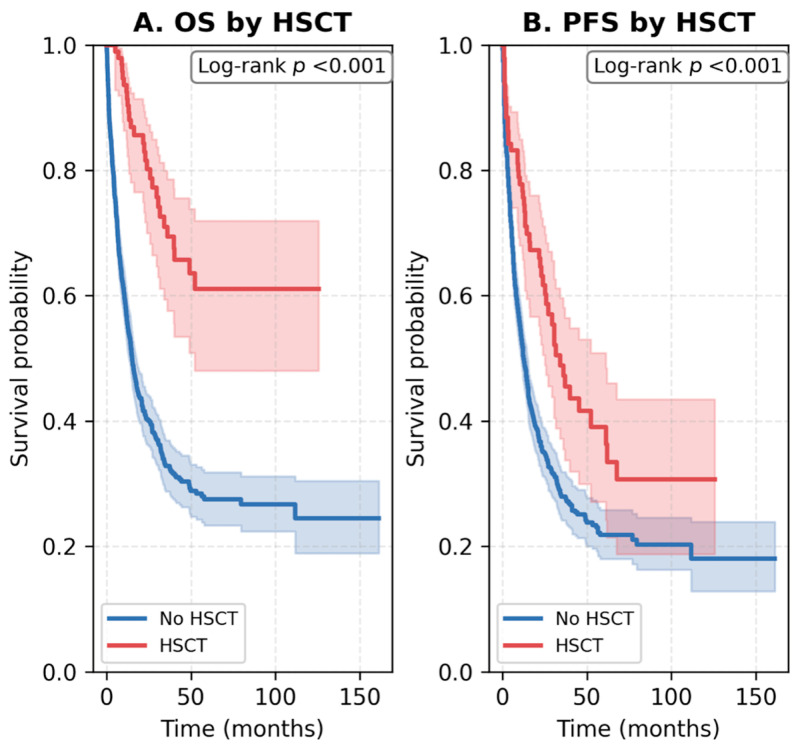
Overall and progression-free survival according to allogeneic HSCT status. (**A**) Kaplan–Meier OS curves comparing patients who underwent allogeneic HSCT versus those who did not. (**B**) Kaplan–Meier PFS curves by HSCT status. Patients receiving HSCT demonstrated markedly improved survival outcomes, with both OS and PFS significantly prolonged compared with the non-HSCT group (log-rank *p* < 0.001 for both analyses). Shaded areas represent 95% confidence intervals. Abbreviations: OS, overall survival; PFS, progression-free survival; HSCT, hematopoietic stem cell transplantation.

The impact of allogeneic HSCT on survival outcomes was analyzed within the study cohort. Among all 891 patients, 99 (11.1%) underwent allogeneic HSCT, predominantly within the high-intensity group (18.9% in 7 + 3 induction), while no HSCT was performed among patients treated with low-intensity regimens. In patients aged ≥60 years, only 15 (3.8%) underwent HSCT, reflecting real-world limitations in transplant eligibility due to age and comorbidities. Transplanted patients achieved markedly improved survival compared with those who did not undergo HSCT. Median OS was not reached in the HSCT group versus 15.1 months in the non-HSCT group (log-rank *p* < 0.001), and median PFS was 34.1 versus 12.6 months, respectively (log-rank *p* < 0.001). These findings are illustrated in Figure 6.

### 3.5. Impact of ECOG Performance Status

**Figure 7:**
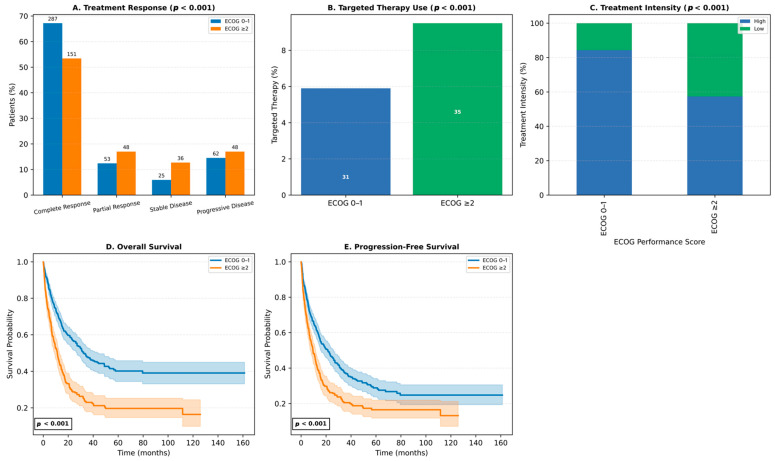
Impact of ECOG performance status on treatment response, targeted therapy use, treatment intensity, and survival outcomes. (**A**) Treatment response distribution (complete remission [CR], partial response [PR], stable disease [SD], progressive disease [PD]) according to ECOG performance status (0–1 vs. ≥2) (*p* < 0.001). (**B**) Proportion of patients receiving targeted therapy stratified by ECOG status (*p* < 0.001). (**C**) Distribution of treatment intensity (high-intensity vs. low-intensity) according to ECOG status (*p* < 0.001). (**D**) Kaplan–Meier curves showing overall survival (OS) stratified by ECOG status (*p* < 0.001). (**E**) Kaplan–Meier curves showing progression-free survival (PFS) stratified by ECOG status (*p* < 0.001). Abbreviations: ECOG, Eastern Cooperative Oncology Group; CR, complete remission; PR, partial response; SD, stable disease; PD, progressive disease; OS, overall survival; PFS, progression-free survival.

Baseline ECOG status was a major determinant of treatment patterns and outcomes. Patients with ECOG 0–1 had higher CR rates (67.2%) and lower PD rates (14.5%) than those with ECOG ≥ 2 (CR: 53.4%, PD: 17.0%) (Figure 7A). Targeted therapies were used more frequently in ECOG ≥ 2 patients (8.7% vs. 5.2%), indicating a shift toward personalized treatment in frailer individuals (Figure 7B). High-intensity regimens were administered to 71.6% of ECOG 0–1 patients, but only 52.0% of those with ECOG ≥ 2, who were more often treated with low-intensity regimens (38.7%) (Figure 7C).

ECOG performance status also correlated with survival. Patients with ECOG 0–1 had superior outcomes, with a 1-year OS rate of 70.3% and median OS of 32.2 months, compared to 47.0% and 11.2 months in the ECOG ≥ 2 group. Similarly, 1-year PFS was 62.6% vs. 42.7%, with median PFS of 21.5 vs. 9.5 months (Figure 7D,E). These results underscore the prognostic significance of ECOG status in guiding treatment intensity and anticipating survival outcomes in AML.

## 4. Discussion

This interim prospective analysis of the Turkish AML Registry presents a comprehensive real-world overview of adult AML treatment strategies and clinical outcomes in a large, multicenter national cohort. The findings highlight how treatment intensity, risk classification, and targeted therapy use influence outcomes in routine practice. Notably, intensive regimens achieved higher remission rates, while the addition of targeted agents substantially improved responses in patients receiving low-intensity therapy.

Our results should be interpreted within the broader context of international real-world evidence. While clinical trials predominantly include younger and fitter patients, population-based registries have consistently shown lower remission and survival rates in unselected cohorts, largely driven by older age, comorbidities, and limited transplant access [26,37]. Such real-world datasets complement clinical trials by reflecting treatment heterogeneity and healthcare disparities encountered in daily practice. National AML registries, including those in Sweden, Denmark, Germany, Korea, and now Turkey, provide valuable insights into how evidence-based recommendations are implemented across diverse healthcare systems [7,27].

The CR rates observed in our cohort are comparable to those reported in clinical trials evaluating novel targeted therapies, suggesting that these treatments can achieve similar efficacy in a real-world setting [30,38,39,40,41,42]. However, several real-world studies have highlighted the limitations of low-intensity regimens in elderly AML patients. A multicenter cohort study involving patients aged 60–75 years reported that intensive chemotherapy was associated with significantly longer OS compared to venetoclax plus hypomethylating agent combinations [43]. Similarly, hypomethylating agents alone yielded modest overall response rates of 15–20%, with median OS ranging from 7.7 to 24.5 months [18,44,45]. In contrast, our cohort demonstrated a 1-year OS of 48.5% in patients aged ≥60 years treated with low-intensity regimens plus targeted therapy, significantly higher than the 31.4% in those without targeted agents. Additionally, retrospective analyses in relapsed/refractory AML have demonstrated that both intensive and non-intensive treatments offer a survival advantage over best supportive care [16].

Age remains one of the strongest prognostic indicators in AML, with population-based data showing a 5-year OS of approximately 58% for patients diagnosed before age 40, decreasing by nearly 10% with each subsequent decade [3,23,46]. In our cohort, age ≥ 60 years, ECOG performance status ≥ 2, and adverse or intermediate ELN risk were associated with reduced OS, consistent with prior population-based studies [3,22,23,47]. Although intensive chemotherapy is typically investigated in younger cohorts and reserved for older individuals deemed fit for aggressive treatment [31], our findings revealed that 74.1% of patients underwent intensive induction despite nearly half (45.5%) being aged 60 years or older. This suggests that clinicians’ assessments of patient fitness and initial treatment decisions for AML in community practices may differ from those in clinical trials among selected patients [24,31,48]. Supporting data from other registry-based subgroup analyses in Turkey have shown consistent CR rates with 7 + 3 induction in newly diagnosed patients, and improved outcomes with azacitidine–venetoclax combinations in unfit patients [49,50].

The diverse outcomes in AML patients highlight the critical role of cytogenetic and molecular markers in identifying high-risk patients for relapse and those who may benefit from personalized treatment approaches. Several mutations either respond to specific therapies or present opportunities for targeted interventions [30,32]. Recently, the United States Food and Drug Administration approved several oral targeted therapies for AML management, guided by specific genetic and molecular profiles [30,31]. These therapies are increasingly being utilized as frontline treatments for cases who are ineligible for intensive chemotherapy, as well as in salvage therapy for relapsed or refractory disease, demonstrating better outcomes compared to previous standards of care [1,29,30,31].

In our cohort, the median OS was 27.2 months, aligning with outcomes reported in recent clinical trials evaluating targeted therapies in AML [38,39,42,51,52]. Interestingly, a single-center, retrospective study of 127 AML patients aged 60–75 at diagnosis found no notable difference in OS between those receiving high-intensity chemotherapy and those treated with low-intensity targeted therapy [30].

It is also important to recognize the contextual relevance of our findings. In developing or middle-income countries, AML outcomes remain significantly inferior to those in high-income settings due to delayed diagnosis, limited access to molecular testing, and uneven availability of novel agents [28,53]. Real-world national registries, such as the Turkish AML Registry, are therefore essential for identifying practice gaps, informing healthcare policy, and guiding the equitable implementation of modern AML therapies.

This interim analysis, with a median follow-up of 12.0 months, highlights the potential impact of extended follow-up on survival outcomes. For instance, data from the Italian Compassionate Use Program reported a 1-year OS rate of 68.6% with an initial follow-up of 11 months. However, the median OS was just 13 months, and the estimated 2-year OS rate significantly declined to 29% after 22 months of follow-up [54,55,56]. While novel targeted agents have improved response rates and survival in AML, they are not considered curative on their own. Allogeneic HSCT remains a key potentially curative option, particularly for patients with ELN intermediate- or adverse-risk disease and those with relapsed or refractory AML [30]. In real-world settings, the proportion of patients undergoing HSCT varies but is generally comparable to or even higher than in clinical trials, especially with increased use of targeted agents such as CPX-351 (liposomal cytarabine-daunorubicin combination) [54,56,57,58].

In this real-world AML registry, 11.1% of patients underwent allogeneic HSCT, predominantly following intensive induction therapy. This rate parallels global real-world data, where HSCT utilization ranges from 18–40% in high-income and 3–6% in low- to middle-income countries [59,60]. Transplanted patients achieved markedly better outcomes, consistent with meta-analyses showing that HSCT in CR significantly improves OS and PFS compared with consolidation chemotherapy [61,62,63]. In older adults, reduced-intensity conditioning offers feasible survival benefits while minimizing toxicity [64,65]. Despite promising responses with venetoclax-based regimens, long-term survival remains superior with HSCT, particularly in high-risk or minimal residual disease (MRD)-positive AML [12,66]. Collectively, these findings affirm the central role of HSCT as a curative consolidation approach in appropriately selected AML patients.

Regional and center-specific variations in patient selection and transplant referral practices strongly influence allogeneic HSCT rates across real-world cohorts. In a subgroup analysis involving patients with newly diagnosed AML from the Turkish AML Registry Project database, allogeneic HSCT was performed in 10.9% of participants treated with idarubicin and 26.2% of those treated with daunorubicin, whereas no patients in the mitoxantrone group proceeded to allogeneic HSCT [49]. These findings highlight the need for more consistent and standardized approaches to transplant eligibility and referral in routine clinical practice.

Receipt of allogeneic HSCT, a well-established determinant of survival in AML, may be limited by limited early access to targeted therapies in certain settings, including our country. This is particularly relevant given the suggested link between these therapies and improved feasibility of undergoing allogeneic HSCT [54,58]. However, previous studies indicate that survival rates are similar for AML patients who undergo induction therapy followed by transplantation, regardless of whether remission is achieved through intensive chemotherapy or targeted treatments [30,67,68,69]. The essential role of transplantation in older adults remains paramount, irrespective of the treatment intensity employed to achieve a response [30,56,70]. According to a recent investigation by the Center for International Blood and Marrow Transplant Research, which evaluated prognostic factors influencing post-transplant outcomes in AML patients aged 60 years or older, chronological age alone does not hinder successful transplantation and should not exclude patients from allogeneic HSCT [71].

Several limitations should be acknowledged. As an interim analysis, the study is limited to 1-year outcomes and does not yet include post-relapse survival or long-term HSCT follow-up. Additionally, performance status and treatment intensity were not randomized, introducing potential selection bias. However, the large sample size, prospective design, and multicenter nature enhance the generalizability and relevance of our findings.

Future research should focus on integrating comprehensive genomic, clinical, and health-system data to refine personalized risk stratification in AML. Expanded national registries with standardized NGS and MRD assessment will be critical to validate the prognostic value of the 2022 ELN classification across age and fitness subgroups. Building on multicenter experience from the Turkish AML Registry, where molecular testing and access to *FLT3*-targeted therapy were heterogeneous, forthcoming phases should emphasize centralized genomic analysis, harmonized MRD monitoring, and systematic evaluation of real-world access barriers [72]. Such efforts will enable predictive modeling to guide treatment intensity and transplant selection, while supporting equitable implementation of novel AML therapies in middle-income healthcare systems.

## 5. Conclusions

In conclusion, this nationwide real-world study offers valuable insights into current AML treatment patterns and survival predictors in Turkey. High-intensity chemotherapy remains the most effective approach for eligible patients, but low-intensity regimens combined with targeted agents provide meaningful benefit in selected subgroups. ECOG performance status and ELN risk classification continue to play pivotal roles in guiding treatment intensity and anticipating prognosis. As the AML therapeutic landscape evolves, real-world evidence from prospective registries will be critical to optimizing personalized care and informing national treatment frameworks.

## Figures and Tables

**Table 1:** Baseline Clinical Characteristics and Demographics of the Patient Cohort (n = 891).

**Age (Year), Median (Min–Max)**	**58 (18–91)**
**Gender, n (%)**	
Female	402 (45.1)
Male	489 (54.9)
**WHO subtype, %**	
AML with recurrent genetic abnormalities	17.6
AML with myelodysplasia-related changes	15.4
Therapy-related myeloid neoplasms	0.8
AML not otherwise specified	55.8
Myeloid sarcoma	0.7
Acute leukemias of ambiguous lineage	9.7
**ELN 2017 risk stratification, n (%)**	
Favorable	169 (24.7)
Intermediate	416 (60.8)
Adverse	99 (14.5)
Missing	207
**Extramedullary involvement, n (%)**	
Yes	38 (4.4)
No	834 (95.6)
Missing	19
**ECOG at diagnosis**	
0–1	524 (63.1)
2–4	306 (36.9)
Missing	61
***FLT3-ITD* mutation status**	
Mutated	62 (15.7)
Unmutated	332 (84.3)
Missing	497
***NPM1* mutation status**	
Mutated	55 (20.1)
Unmutated	219 (79.9)
Missing	617

WHO: World Health Organization; ELN: European LeukemiaNet; *FLT3-ITD*: fms-like tyrosine kinase 3-internal tandem duplication; *NPM1*: Nucleophosmin 1.

## Data Availability

Data available on request from the authors.

## Supplementary Materials

The following supporting information can be downloaded at: https://www.mdpi.com/article/10.3390/jcm14207367/s1, Table S1. Frequency and distribution of molecular and cytogenetic alterations among tested AML patients in the Turkish AML Registry. Figure S1. Distribution of targeted therapy agents administered according to treatment intensity. Bar chart illustrating the number of patients receiving specific targeted agents—venetoclax, gemtuzumab ozogamicin, midostaurin, sorafenib, or others—stratified by treatment intensity (high-intensity vs. low-intensity regimens).

## References

[1] DiNardo C.D., Erba H.P., Freeman S.D., Wei A.H. (2023). Acute myeloid leukaemia. Lancet.

[2] Shimony S., Stahl M., Stone R.M. (2023). Acute myeloid leukemia: 2023 update on diagnosis, risk-stratification, and management. Am. J. Hematol..

[3] National Cancer Institute SEER Cancer Stat Facts: Acute Myeloid Leukemia. https://seer.cancer.gov/statfacts/html/amyl.html.

[4] Talati C., Sweet K. (2018). Recently approved therapies in acute myeloid leukemia: A complex treatment landscape. Leuk. Res..

[5] Chen P., Liu X., Zhao Y., Hu Y., Guo J., Wang H. (2024). Global, national, and regional burden of acute myeloid leukemia among 60–89 years-old individuals: Insights from a study covering the period 1990 to 2019. Front. Public Health.

[6] Yi M., Li A., Zhou L., Chu Q., Song Y., Wu K. (2020). The global burden and attributable risk factor analysis of acute myeloid leukemia in 195 countries and territories from 1990 to 2017: Estimates based on the global burden of disease study 2017. J. Hematol. Oncol..

[7] Han H.J., Choi K., Suh H.S. (2024). Impact of aging on acute myeloid leukemia epidemiology and survival outcomes: A real-world, population-based longitudinal cohort study. PLoS ONE.

[8] Pollyea D.A., Altman J.K., Assi R., Bixby D., Fathi A.T., Foran J.M., Gojo I., Hall A.C., Jonas B.A., Kishtagari A. (2023). Acute myeloid leukemia, version 3.2023, NCCN clinical practice guidelines in oncology. J. Natl. Compr. Cancer Netw..

[9] Burnett A.K., Milligan D., Prentice A.G., Goldstone A.H., McMullin M.F., Hills R.K., Wheatley K. (2007). A comparison of low-dose cytarabine and hydroxyurea with or without all-trans retinoic acid for acute myeloid leukemia and high-risk myelodysplastic syndrome in patients not considered fit for intensive treatment. Cancer Interdiscip. Int. J. Am. Cancer Soc..

[10] Bittar G., De Oliveira-Gomes D., Rivero G. (2022). Advances and Future Goals in Acute Myeloid Leukaemia Therapy. Oncol. Haematol..

[11] Meillon-Garcia L.A., Demichelis-Gómez R. (2020). Access to Therapy for Acute Myeloid Leukemia in the Developing World: Barriers and Solutions. Curr. Oncol. Rep..

[12] Bhansali R.S., Pratz K.W., Lai C. (2023). Recent advances in targeted therapies in acute myeloid leukemia. J. Hematol. Oncol..

[13] Bazinet A., Kantarjian H., Arani N., Popat U., Bataller A., Sasaki K., DiNardo C.D., Daver N., Yilmaz M., Abbas H.A. (2023). Evolving trends and outcomes in older patients with acute myeloid leukemia including allogeneic stem cell transplantation. Am. J. Hematol..

[14] Kantarjian H., Borthakur G., Daver N., DiNardo C.D., Issa G., Jabbour E., Kadia T., Sasaki K., Short N.J., Yilmaz M. (2024). Current status and research directions in acute myeloid leukemia. Blood Cancer J..

[15] O’Donnell M.R., Abboud C.N., Altman J., Appelbaum F.R., Arber D.A., Attar E., Borate U., Coutre S.E., Damon L.E., Goorha S. (2012). Acute myeloid leukemia. J. Natl. Compr. Cancer Netw..

[16] Brandwein J.M., Saini L., Geddes M.N., Yusuf D., Liu F., Schwann K., Billawala A., Westcott C., Kurniawan J.A., Cheung W.Y. (2020). Outcomes of patients with relapsed or refractory acute myeloid leukemia: A population-based real-world study. Am. J. Blood Res..

[17] Dombret H., Seymour J.F., Butrym A., Wierzbowska A., Selleslag D., Jang J.H., Kumar R., Cavenagh J., Schuh A.C., Candoni A. (2015). International phase 3 study of azacitidine vs conventional care regimens in older patients with newly diagnosed AML with >30% blasts. Blood J. Am. Soc. Hematol..

[18] Kantarjian H.M., Thomas X.G., Dmoszynska A., Wierzbowska A., Mazur G., Mayer J., Gau J.-P., Chou W.-C., Buckstein R., Cermak J. (2012). Multicenter, randomized, open-label, phase III trial of decitabine versus patient choice, with physician advice, of either supportive care or low-dose cytarabine for the treatment of older patients with newly diagnosed acute myeloid leukemia. J. Clin. Oncol..

[19] Döhner H., Estey E., Grimwade D., Amadori S., Appelbaum F.R., Büchner T., Dombret H., Ebert B.L., Fenaux P., Larson R.A. (2017). Diagnosis and management of AML in adults: 2017 ELN recommendations from an international expert panel. Blood J. Am. Soc. Hematol..

[20] Liesveld J. (2012). Management of AML: Who do we really cure?. Leuk. Res..

[21] Ciftciler R., Demiroglu H., Haznedaroglu I.C., Sayınalp N., Aksu S., Ozcebe O., Goker H., Aydın M.S., Buyukasık Y. (2019). Impact of time between induction chemotherapy and complete remission on survival outcomes in patients with acute myeloid leukemia. Clin. Lymphoma Myeloma Leuk..

[22] Wang C.-Y., Huang H.-H., Chen H.-M., Hsiao F.-Y., Ko B.-S. (2021). Real-world outcomes of patients with acute myeloid leukemia in Taiwan: A nationwide population-based study, 2011–2015. Clin. Lymphoma Myeloma Leuk..

[23] Shallis R.M., Boddu P.C., Bewersdorf J.P., Zeidan A.M. (2020). The golden age for patients in their golden years: The progressive upheaval of age and the treatment of newly-diagnosed acute myeloid leukemia. Blood Rev..

[24] Jaramillo Segura S., Schlenk R.F. (2023). Update on current treatments for adult acute myeloid leukemia. Haematologica.

[25] Heuser M., Fernandez C., Hauch O., Klibanov O.M., Chaudhary T., Rives V. (2023). Therapies for acute myeloid leukemia in patients ineligible for standard induction chemotherapy: A systematic review. Future Oncol..

[26] Juliusson G., Lazarevic V., Hörstedt A.-S., Hagberg O., Höglund M., Swedish Acute Leukemia Registry Group (2012). Acute myeloid leukemia in the real world: Why population-based registries are needed. Blood.

[27] Østgård L.S., Nørgaard J.M., Raaschou-Jensen K.K., Pedersen R.S., Rønnov-Jessen D., Pedersen P.T., Dufva I.H., Marcher C.W., Nielsen O.J., Severinsen M.T. (2016). The Danish National Acute Leukemia Registry. Clin. Epidemiol..

[28] Campos C., Orathes Ponte Silva A.M., Feistauer F., Teixeira Da Silva L., Favano T., Salvino M.A. (2024). 10-Year Real-World Data on Acute Myeloid Leukemia. J. Bone Marrow Transplant. Cell. Ther..

[29] Burd A., Levine R.L., Ruppert A.S., Mims A.S., Borate U., Stein E.M., Patel P.A., Baer M.R., Stock W., Deininger M.W. (2019). Precision medicine treatment in older AML: Results of Beat AML Master Trial. Blood.

[30] Sahasrabudhe K., Huang Y., Rebechi M., Elder P., Mims A., Wall S. (2022). Survival, response rates, and post-transplant outcomes in patients with acute myeloid leukemia aged 60–75 treated with high intensity chemotherapy vs. lower intensity targeted therapy. Front. Oncol..

[31] Kantarjian H.M., Kadia T.M., DiNardo C.D., Welch M.A., Ravandi F. (2021). Acute myeloid leukemia: Treatment and research outlook for 2021 and the MD Anderson approach. Cancer.

[32] Bhatt V.R. (2021). Advances and unanswered questions in management of acute myeloid leukemia in older adults: A glimpse into the future. J. Geriatr. Oncol..

[33] Göker H., Çınar O.E., Demiroğlu H., Malkan Ü.Y., Aladağ Karakulak E., Büyükaşık Y. (2023). Venetoclax and Azacitidine Treatment in Relapsed Acute Myeloid Leukemia after Hematopoietic Stem Cell Transplantation: A Cohort Study in the Real-World Setting of a Tertiary Center. Turk. J. Hematol..

[34] Gemici A., Ozkalemkas F., Dogu M.H., Tekinalp A., Alacacioglu I., Guney T., Ince I., Geduk A., Cagliyan G.A., Maral S. (2021). A Real-life Turkish Experience of Venetoclax Treatment in High-risk Myelodysplastic Syndrome and Acute Myeloid Leukemia. Clin. Lymphoma Myeloma Leuk..

[35] American Cancer Society Acute Myeloid Leukemia (AML) Subtypes and Prognostic Factors. https://www.cancer.org/cancer/types/acute-myeloid-leukemia/detection-diagnosis-staging/how-classified.html.

[36] Bataller Torralba A., Garrido A., Guijarro F., Oñate G., Díaz-Beyá M., Arnan M., Salamero O. (2022). European LeukemiaNet 2017 risk stratification for acute myeloid leukemia: Validation in a risk-adapted protocol. Blood Adv..

[37] Marshalek J.P., Epistola R., Tomassetti S. (2025). Real-world treatment outcomes from a retrospective cohort of patients with acute myeloid leukemia from an urban safety net hospital. J. Oncol. Pharm. Pract..

[38] Perl A.E., Martinelli G., Cortes J.E., Neubauer A., Berman E., Paolini S., Montesinos P., Baer M.R., Larson R.A., Ustun C. (2019). Gilteritinib or Chemotherapy for Relapsed or Refractory *FLT3*-Mutated AML. N. Engl. J. Med..

[39] Wang E.S., Montesinos P., Minden M.D., Lee J.-H., Heuser M., Naoe T., Chou W.-C., Laribi K., Esteve J., Altman J.K. (2022). Phase 3 trial of gilteritinib plus azacitidine vs azacitidine for newly diagnosed *FLT3*^mut+^ AML ineligible for intensive chemotherapy. Blood.

[40] DiNardo C.D., Schuh A.C., Stein E.M., Montesinos P., Wei A.H., de Botton S., Zeidan A.M., Fathi A.T., Kantarjian H.M., Bennett J.M. (2021). Enasidenib plus azacitidine versus azacitidine alone in patients with newly diagnosed, mutant-IDH2 acute myeloid leukaemia (AG221-AML-005): A single-arm, phase 1b and randomised, phase 2 trial. Lancet Oncol..

[41] DiNardo C.D., Jonas B.A., Pullarkat V., Thirman M.J., Garcia J.S., Wei A.H., Konopleva M., Döhner H., Letai A., Fenaux P. (2020). Azacitidine and venetoclax in previously untreated acute myeloid leukemia. N. Engl. J. Med..

[42] Wang E.S., Montesinos P., Minden M.D., Lee J.H., Heuser M., Naoe T., Chou W.C., Laribi K., Esteve J., Altman J.K. (2021). Phase 3, Open-Label, Randomized Study of Gilteritinib and Azacitidine Vs Azacitidine for Newly Diagnosed-Mutated Acute Myeloid Leukemia in Patients Ineligible for Intensive Induction Chemotherapy. Blood.

[43] Matthews A.H., Perl A.E., Luger S.M., Gill S.I., Lai C., Porter D.L., Skuli S., Bruno X.J., Carroll M.P., Freyer C.W. (2023). Real-world effectiveness of intensive chemotherapy with 7&3 versus venetoclax and hypomethylating agent in acute myeloid leukemia. Am. J. Hematol..

[44] Gore S.D., Fenaux P., Santini V., Bennett J.M., Silverman L.R., Seymour J.F., Hellström-Lindberg E., Swern A.S., Beach C.L., List A.F. (2013). A multivariate analysis of the relationship between response and survival among patients with higher-risk myelodysplastic syndromes treated within azacitidine or conventional care regimens in the randomized AZA-001 trial. Haematologica.

[45] He F., Sapkota S., Parker S., Defor T., Warlick E., Ustun C., Eckfeldt C., Rashidi A., Kurtzweil A., Weisdorf D. (2018). A real-world study of clofarabine and cytarabine combination therapy for patients with acute myeloid leukemia. Leuk. Lymphoma.

[46] Shallis R.M., Wang R., Davidoff A., Ma X., Zeidan A.M. (2019). Epidemiology of acute myeloid leukemia: Recent progress and enduring challenges. Blood Rev..

[47] Song X., Peng Y., Wang X., Chen Y., Jin L., Yang T., Qian M., Ni W., Tong X., Lan J. (2018). Incidence, survival, and risk factors for adults with acute myeloid leukemia not otherwise specified and acute myeloid leukemia with recurrent genetic abnormalities: Analysis of the surveillance, epidemiology, and end results (SEER) database, 2001–2013. Acta Haematol..

[48] Sasaki K., Ravandi F., Kadia T.M., DiNardo C.D., Short N.J., Borthakur G., Jabbour E., Kantarjian H.M. (2021). De novo acute myeloid leukemia: A population-based study of outcome in the United States based on the Surveillance, Epidemiology, and End Results (SEER) database, 1980 to 2017. Cancer.

[49] Pinar I.E., Celik S., Polat M.G., Karatas A.F., Dogan A., Iltar U., Seval G.C., Malkan U.Y., Ince I., Yenihayat E.M. (2023). Idarubicin Versus Daunorubicin Versus Mitoxantrone for Induction Chemotherapy in Acute Myeloid Leukemia: Patient Registration Study of Turkish Society of Hematology-Acute Myeloid Leukemia Working Group. Blood.

[50] Urlu S.M., Cengiz Seval G., Erdogan Yücel E., Mehtap O., Yenihayat E.M., Polat M.G., Malkan U.Y., Ozbalci D., Yigit Kaya S., Durusoy S.S. (2023). Acute Myeloid Leukemia in Elderly, Unfit Patients: Analysis of Turkish AML Prospective Registry Database, on Behalf of Acute Leukemia Working Group of Turkish Society of Hematology. Blood.

[51] Dinardo C.D., Stein E.M., De Botton S., Roboz G.J., Altman J.K., Mims A.S., Swords R., Collins R.H., Mannis G.N., Pollyea D.A. (2018). Durable Remissions with Ivosidenib in *IDH1*-Mutated Relapsed or Refractory AML. N. Engl. J. Med..

[52] Montesinos P., Recher C., Vives S., Zarzycka E., Wang J., Bertani G., Heuser M., Calado R.T., Schuh A.C., Yeh S.-P. (2022). Ivosidenib and Azacitidine in *IDH1*-Mutated Acute Myeloid Leukemia. N. Engl. J. Med..

[53] Van Weelderen R.E., Klein K., Natawidjaja M.D., De Vries R., Kaspers G.J. (2021). Outcome of pediatric acute myeloid leukemia (AML) in low- and middle-income countries: A systematic review of the literature. Expert. Rev. Anticancer. Ther..

[54] Guolo F., Fianchi L., Minetto P., Clavio M., Gottardi M., Galimberti S., Rizzuto G., Rondoni M., Bertani G., Dargenio M. (2020). CPX-351 treatment in secondary acute myeloblastic leukemia is effective and improves the feasibility of allogeneic stem cell transplantation: Results of the Italian compassionate use program. Blood Cancer J..

[55] Minetto P., Guolo F., Fianchi L., Clavio M., Gottardi M., Endri M., Galimberti S., Rizzuto G., Sciumé M., Rondoni M. (2021). CPX-351 Induction in Secondary Acute Myeloblastic Leukemia: Extended Follow up from the Italian Compassionate Use Program. Blood.

[56] Lemoli R.M., Montesinos P., Jain A. (2023). Real-world experience with CPX-351 in high-risk acute myeloid leukemia. Crit. Rev. Oncol./Hematol..

[57] Lancet J.E., Uy G.L., Newell L.F., Lin T.L., Ritchie E.K., Stuart R.K., Strickland S.A., Hogge D., Solomon S.R., Bixby D.L. (2021). CPX-351 versus 7+ 3 cytarabine and daunorubicin chemotherapy in older adults with newly diagnosed high-risk or secondary acute myeloid leukaemia: 5-year results of a randomised, open-label, multicentre, phase 3 trial. Lancet Haematol..

[58] Matthews A.H., Perl A.E., Luger S.M., Loren A.W., Gill S.I., Porter D.L., Babushok D.V., Maillard I.P., Carroll M.P., Frey N.V. (2022). Real-world effectiveness of CPX-351 vs venetoclax and azacitidine in acute myeloid leukemia. Blood Adv..

[59] Rashidi A., Ebadi M., Colditz G.A., DiPersio J.F. (2016). Outcomes of Allogeneic Stem Cell Transplantation in Elderly Patients with Acute Myeloid Leukemia: A Systematic Review and Meta-analysis. Biol. Blood Marrow Transplant..

[60] Tokaz M.C., Baldomero H., Cowan A.J., Saber W., Greinix H., Koh M.B.C., Kröger N., Mohty M., Galeano S., Okamoto S. (2023). An Analysis of the Worldwide Utilization of Hematopoietic Stem Cell Transplantation for Acute Myeloid Leukemia. Transplant. Cell. Ther..

[61] Begna K.H., Kittur J., Gangat N., Alkhateeb H., Patnaik M.S., Al-Kali A., Elliott M.A., Hogan W.J., Litzow M.R., Pardanani A. (2022). European LeukemiaNet-defined primary refractory acute myeloid leukemia: The value of allogeneic hematopoietic stem cell transplant and overall response. Blood Cancer J..

[62] Masetti R., Muratore E., Gori D., Prete A., Locatelli F. (2022). Allogeneic hematopoietic stem cell transplantation for pediatric acute myeloid leukemia in first complete remission: A meta-analysis. Ann. Hematol..

[63] Servais S., Beguin Y., Baron F. (2022). Current Status and Perspectives of Allogeneic Hematopoietic Stem Cell Transplantation in Elderly Patients with Acute Myeloid Leukemia. Stem Cells Transl. Med..

[64] Backhaus D., Brauer D., Pointner R., Bischof L., Vucinic V., Franke G.-N., Niederwieser D., Platzbecker U., Jentzsch M., Schwind S. (2023). A high hematopoietic cell transplantation comorbidity Index (HCT-CI) does not impair outcomes after non-myeloablative allogeneic stem cell transplantation in acute myeloid leukemia patients 60 years or older. Bone Marrow Transplant..

[65] Zhang Z.-H., Lian X.-Y., Yao D.-M., He P.-F., Ma J.-C., Xu Z.-J., Guo H., Zhang W., Lin J., Qian J. (2017). Reduced intensity conditioning of allogeneic hematopoietic stem cell transplantation for myelodysplastic syndrome and acute myeloid leukemia in patients older than 50 years of age: A systematic review and meta-analysis. J. Cancer Res. Clin. Oncol..

[66] Mohty R., El Hamed R., Brissot E., Bazarbachi A., Mohty M. (2023). New drugs before, during, and after hematopoietic stem cell transplantation for patients with acute myeloid leukemia. Haematologica.

[67] Perl A.E., Larson R.A., Podoltsev N.A., Strickland S., Wang E.S., Schiller G.J., Martinelli G., Neubauer A., Sierra J., Montesinos P. (2021). Follow-up of patients with FLT3-mutated R/R AML in the phase 3 ADMIRAL trial. J. Clin. Oncol..

[68] Pratz K.W., DiNardo C.D., Arellano M.L., Letai A.G., Thirman M., Pullarkat V.A., Roboz G.J., Becker P.S., Hong W.-J., Jiang Q. (2019). Outcomes after stem cell transplant in older patients with acute myeloid leukemia treated with venetoclax-based therapies. Blood.

[69] Kennedy V.E., Hui G., Gaut D., Mittal V., Oliai C., Muffly L.S., Logan A.C., Mannis G.N. (2020). Hypomethylating agents in combination with venetoclax as a bridge to allogeneic transplant in acute myeloid leukemia. Blood.

[70] Gupta V., Tallman M.S., Weisdorf D.J. (2011). Allogeneic hematopoietic cell transplantation for adults with acute myeloid leukemia: Myths, controversies, and unknowns. Blood J. Am. Soc. Hematol..

[71] Maakaron J.E., Zhang M.-J., Chen K., Abhyankar S., Bhatt V.R., Chhabra S., El Jurdi N., Farag S.S., He F., Juckett M. (2022). Age is no barrier for adults undergoing HCT for AML in CR1: Contemporary CIBMTR analysis. Bone Marrow Transplant..

[72] Pinar I.E., Celik S., Polat M.G., Karatas A.F., Dogan A., Iltar U., Cengiz Seval G., Malkan U.Y., Ince I., Yenihayat E.M. (2025). Comprehensive analysis of FLT3-mutated patients with acute myeloid leukemia with updated 2022 European LeukemiaNet recommendations: Insights from the Turkish AML registry project. BMC Cancer.

