# Supplementary material for: Clinical Characteristics, Treatment Approaches, and Survival Predictors in Adult Acute Myeloid Leukemia: Interim Results from the Turkish Society of Hematology AML Registry
Source: J Clin Med. 2025 Oct 18;14(20):7367. doi: 10.3390/jcm14207367 (PMC12565015; doi:10.3390/jcm14207367)
Supplement: Supplementary file 1 [file jcm-14-07367-s001.zip › jcm-3889713-supplementary.pdf]

Supplementary Table S1. Frequency and distribution of molecular and cytogenetic alterations among tested AML patients in the Turkish AML Registry

Gene / abnormality	Mutated, n	Mutated, %	Wild-type, n	Wild-type, %	Missing, n	Tested (n)	Testing method(s)
FLT3-ITD	62	15.7	332	84.3	497	394	PCR / NGS
FLT3-TKD	10	5.3	179	94.7	702	189	PCR / NGS
NPM1	55	20.1	219	79.9	617	274	PCR / NGS
IDH1/2	5	5.9	80	94.1	806	85	PCR / NGS
del(17p) / *TP53* alteration	7	7.4	87	92.6	797	94	FISH / Cytogenetic (narrative extraction) / PCR / NGS
RUNX1	7	5.7	115	94.3	769	122	PCR / NGS
ASXL1	0	0.0	23	100.0	868	23	PCR / NGS
CEBPA	3	4.8	59	95.2	829	62	PCR / NGS
C-KIT	22	22.4	76	77.6	793	98	PCR / NGS
t(15;17)/PML-RARA	72	11.7	546	88.3	273	618	PCR / NGS / FISH
t(8;21)	49	8.5	529	91.5	313	578	NGS / FISH
inv(16)/t(16;16)	13	3.1	409	96.9	469	422	NGS / FISH
inv(3)/t(3;3)	3	2.3	126	97.7	762	129	NGS / FISH
t(6;9)	1	1.8	55	98.2	835	56	NGS / FISH
t(9;22)/BCR-ABL1	10	1.8	552	98.2	329	562	PCR / NGS / FISH
KMT2A (11q23) rearrangement	15	3.8	380	96.2	496	395	PCR / NGS / FISH

Frequencies are reported among tested patients for each marker; percentages are calculated as Mutated / Tested × 100. Testing methods reflect site availability (PCR, NGS, FISH). Missing = untested or unavailable results. The availability of molecular and cytogenetic testing varied widely across participating centers. FLT3 and NPM1 were the most frequently assessed markers, while other genes (IDH1/2, TP53, RUNX1, ASXL1, CEBPA, KIT, and KMT2A) were analyzed only in centers equipped with PCR or NGS facilities. Cytogenetic abnormalities, including recurrent translocations and deletions such as del(17p), were identified through conventional karyotyping, FISH, or narrative cytogenetic reports. Missing data were not imputed; ELN 2017 risk categories were assigned according to the available molecular and cytogenetic information for each patient. This heterogeneity reflects the real-world diagnostic landscape during the registry period and underscores the study's prospective, practice-based design.

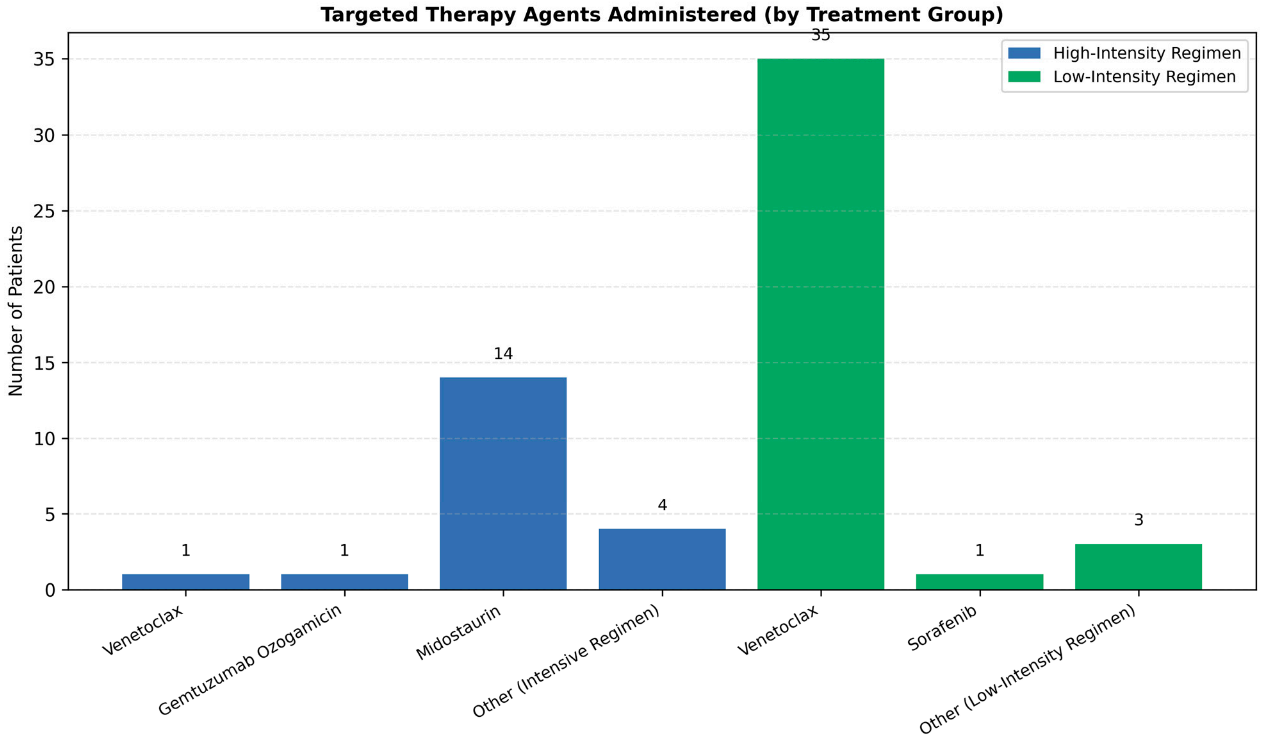

Supplementary Figure S1. Distribution of targeted therapy agents administered according to treatment intensity. *Bar chart illustrating the number of patients receiving specific targeted agents—venetoclax, gemtuzumab ozogamicin, midostaurin, sorafenib, or others—stratified by treatment intensity (high-intensity vs. low-intensity regimens).*
